# Renal Infiltration of Follicular Lymphoma

**DOI:** 10.4274/tjh.2014.0013

**Published:** 2014-09-05

**Authors:** Ivan Petković, Miljan Krstić, Ivica Pejcić, Svetislav Vrbić, Slavica Stojnev, Ana Cvetanović, Mirjana Balić, Mirjana Todorović

**Affiliations:** 1 Niš University Clinical Center, Clinic of Oncology, Department of Hematological Malignancies, Niš, Serbia; 2 Niš University Clinical Center, Institute of Pathology, Niš, Serbia; 3 Niš University Faculty of Medicine, Niš, Serbia

**Keywords:** Primary renal lymphoma, Follicular lymphoma, Treatment option

## TO THE EDITOR

We present a case of renal infiltration of grade 3A follicular lymphoma (FL) mimicking renal cell carcinoma (RCC).

**Figure 1:**
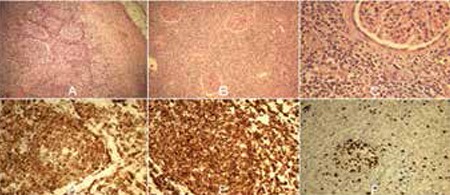
A, B, C) Representative H&E stain photomicrographs of the kidney with grade 3A FL: A) typical follicular growth pattern in the kidney with FL, 40x; B) glomeruli immersed in diffuse lymphomatous mass of FL, 100x; C) glomerulus and renal diffuse lymphoma infiltration, 400x. D, E, F) Immunohistochemical staining representative for FL, 200x: D) diffuse positivity to CD20; E) intense staining of germinal center of neoplastic follicle with Bcl-2; F) Ki-67 proliferative activity (about20%), 339x169 mm (72x72 DPI).

Chronic renal failure (CRF) was diagnosed in a 63-year-old male during routine health controls. Ultrasonography results emphasized a left renal mass. Multislice computed tomography (MSCT) confirmed the prior findings of a solid multilobular mass (48x46x79 mm), reduced kidney parenchyma, and intact surrounding lymph nodes (LNs) mimicking RCC. Without previous oncological evaluation, urologists performed nephrectomy. Histopathology revealed FL of grade 3A, with perirenal infiltration. Immunohistochemistry was typical: CD79α+, CD20+, CD10+, MUM1-, Bcl-2+, Bcl-6+, cyclin D1-, CD23-, CD3, CD5-, CK AE1/AE3-, and EMA-, with a Ki-67 index of 20% (Figure 1). After surgery, the patient underwent a lymphoma staging procedure at our clinic. MSCT scans, bone marrow biopsy, and blood analysis results were within normal ranges. Only residual CRF was maintained and β2-microglobulin level was 6.2 mg/L. The performed PET/CT detected left-sided paravertebral (Th4, Th5) and axilla LNs [standardized uptake value (SUV) max: 7.4]. Since it was high-grade advanced FL with a Follicular International Prognostic Index (FLIPI) score of 2, we conducted 8 R-CHOP induction cycles (left ventricular ejection fraction: 70%). Posttreatment PET/CT verified complete response. Informed consent was obtained from the patient.

Primary renal lymphoma (PRL) is defined as lymphoma arising in the renal parenchyma and not invasion from an adjacent lymphomatous mass [1]. Since the kidney is not a lymphoid organ, proposed mechanisms may include dissemination from subcapsular lymphatics, retroperitoneal lymphoma extension, or lymphoplasmacytic infiltrative inflammatory kidney disease [2]. PRL may be associated with some acute or chronic diseases (chronic pyelonephritis, Sjögren’s syndrome, systemic erythematous lupus, or Epstein–Barr virus infection) [3].

PRL is a systemic disease manifesting initially in the kidneys, with poor outcome even if localized. It disseminates rapidly and mean survival is less than 1 year after diagnosis [4]. Renal involvement is seen in disseminated disease or in relapse. PRL is mostly of diffuse large B-cell lymphoma (DLBCL) type, while FL is exceptionally unusual, with scarce literature data.

Standardized PRL treatment is undefined. R-CHOP could be the elective treatment according to experience and some recently published studies [5,6]. Improved overall survival (OS) in FL with the addition of rituximab to conventional chemotherapy is well documented. High-grade FL and SUV were our major arguments for applying anthracyclines. We expected a better response rate (OS not improved) and reduced risk of FL transformation into DLBCL. The patient’s transforming status was speculated. Cardiotoxicity (early/delayed) and salvage therapy selection were the opposing arguments.

Long-term survivors occasionally have been reported after combined complete resection surgery/chemotherapy treatment, with longer disease-free survival and OS, but only one with kidney involvement [1,7]. The above-mentioned option is not the standard treatment. We advise that solid renal masses undergo biopsy and teamwork consideration (oncologists, radiologists, urologists) prior to eventual nephrectomy decisions.

In conclusion, solid renal lesions mimicking RCC may suggest underlying renal lymphoma infiltration. Although exceptionally uncommon, renal FL may be the pathological subtype. No standardized therapy exists in this specific situation. Rituximab with its documented survival benefit in FL is recommended to be included in the treatment regimen. The accompanying chemotherapy may depend on many variables, including ECOG performance status, patient age, FL grade, FLIPI index, and single-center decision.

## CONFLICT OF INTEREST STATEMENT

The authors of this paper have no conflicts of interest, including specific financial interests, relationships, and/ or affiliations relevant to the subject matter or materials included.

## References

[1] Okuno SH, Hoyer JD, Ristow K, Witzig TE (1995). Primary renal non-Hodgkin’s lymphoma. An unusual extranodal site. Cancer.

[2] Omer HA, Hussein MR (2007). Primary renal lymphoma. Nephrology (Carlton).

[3] Stallone G, Infante B, Manno C, Campobasso N, Pannarale G, Schena FP (2000). Primary renal lymphoma does exist: case report and review of the literature. J Nephrol.

[4] Skarin A (2003). Uncommon presentation of non-Hodgkin’s lymphoma. Case 3. Primary renal lymphoma. J Clin Oncol.

[5] Belbaraka R, Elyoubi MB, Boutayeb S, Errihani H (2011). Primary renal non-Hodgkin lymphoma: an unusual diagnosis for a renal mass. Indian J Cancer.

[6] Vazquez Alonso F, Sanchez Ramos C, Vicente Prados FJ, Pascual Geler M, Ruiz Carazo E, Becerra Massare P, Funes Padilla C, Rodriguez Herrera F, Cozar Olmo JM, Tallada Bunuel M (2009). Primary renal lymphoma: report of three new cases and literature review. Arch Esp Urol.

[7] Cupisti A, Riccioni R, Carulli G, Paoletti S, Tognetti A, Meola M, Francesca F, Barsotti G, Petrini M (2004). Bilateral primary renal lymphoma treated by surgery and chemotherapy. Nephrol Dial Transplant.

